# Novel preclinical murine model of trauma-induced elbow stiffness

**DOI:** 10.1186/s40634-018-0155-3

**Published:** 2018-09-18

**Authors:** Stephanie N. Moore-Lotridge, William K. Oelsner, Yael Ihejirika, Mihir J. Desai, Sandra S. Gebhart, Jonathan G. Schoenecker

**Affiliations:** 1 Department of Orthopaedics and Rehabilitation, Vanderbilt University Medical Center, 1215 21st Ave. South, Suite 4200 MCE, South Tower, Nashville, TN 37232 USA; 2 Department of Pathology Microbiology and Immunology, Vanderbilt University Medical Center, 1161 21st Ave. South, Nashville, TN 37232 USA; 3 Department of Pediatrics, Vanderbilt University Medical Center, 4202 Doctor’s Office Tower, 2200 Children’s Way, Nashville, TN 37232 USA; 4 Department of Pharmacology, Vanderbilt University Medical Center, 2200 Pierce Ave, Robinson Research Building, Rm 454, Nashville, TN 37232 USA

**Keywords:** Elbow stiffness, Murine model, Plasminogen, Plasmin, Preclinical model, Elbow trauma

## Abstract

**Background:**

Peri-articular injury may result in functional deficits and pain. In particular, post-traumatic elbow stiffness is a debilitating condition, precluding patients from performing activities of daily living. As such, clinicians and basic scientists alike, aim to develop novel therapeutic interventions to prevent and treat elbow stiffness; thereby reducing patient morbidity. Yet, there is a paucity of pre-clinical models of peri-articular stiffness, especially of the upper extremity, necessary to develop and test the efficacy of therapeutics. We set out to develop a pre-clinical murine model of elbow stiffness, resulting from soft tissue injury, with features characteristic of pathology observed in these patients.

**Methods:**

A soft tissue peri-elbow injury was inflicted in mice using cardiotoxin. Pathologic tissue repair was induced by creating an investigator-imposed deficiency of plasminogen, a protease essential for musculoskeletal tissue repair. Functional testing was conducted through analysis of grip strength and gait. Radiography, microcomputed tomography, and histological analyses were employed to quantify development of heterotopic ossification.

**Results:**

Animals with peri-elbow soft tissues injury in conjunction with an investigator-imposed plasminogen deficiency, developed a significant loss of elbow function measured by grip strength (2.387 ± 0.136 N vs 1.921 ± 0.157 N, ****, *p* < 0.0001) and gait analysis (35.05 ± 2.775 mm vs 29.87 ± 2.075 mm, ***, *p* < 0.0002). Additionally, plasminogen deficient animals developed capsule thickening, delayed skeletal muscle repair, fibrosis, chronic inflammation, and heterotopic ossification; all features characteristic of pathology observed in patients with trauma-induced elbow stiffness.

**Conclusion:**

A soft tissue injury to the peri-elbow soft tissue with a concomitant deficiency in plasminogen, instigates elbow stiffness and pathologic features similar to those observed in humans. This pre-clinical model is valuable for translational studies designed to investigate the contributions of pathologic features to elbow stiffness or as a high-throughput model for testing therapeutic strategies designed to prevent and treat trauma-induced elbow stiffness.

**Electronic supplementary material:**

The online version of this article (10.1186/s40634-018-0155-3) contains supplementary material, which is available to authorized users.

## Background

Elbow stiffness following peri-articular injury is a debilitating condition, precluding patients from performing activities from daily living. In order for individuals to perform 90% of their daily activities, such as bathing and eating independently, an arc of elbow motion of 100 degrees (30° extension to 130° flexion; 50° pronation to 50° supination) is necessary (Agarwal et al. 2017). Furthermore, it has been reported that a decrease in arc of elbow motion of only 50%, results in an 80% loss of elbow function (Morrey et al. 1981). As such, clinicians and basic scientists alike, aim to develop novel therapeutic interventions to prevent and treat elbow stiffness; thereby reducing patient morbidity. However, to date, no murine models have been developed which recapitulate the soft tissue pathology (fibrosis, capsular thickening, or heterotopic ossification) or the functional deficits (elbow stiffness) seen in patients clinically.

Elbow stiffness can arise following either a local trauma to the elbow (Evans et al. 2009; Josefsson et al. 1984; Weiss and Sachar 1994) or in conjunction with an anatomically remote severe trauma such as burns or head injuries (Brinsden et al. 2008; Djurickovic et al. 1996; Garland and O’Hollaren 1982; Seth and Khurana 1985). Correspondingly, for more than 200 years it has been recognized that severe trauma provokes systemic changes throughout the body. One such response to severe trauma is the dysregulation of plasmin, the main protease of the fibrinolytic system. As such, severely injured patients can experience both over exuberant generation of plasmin, leading to hyperfibrinolysis that portends to death form bleeding, or prolonged deficit of plasmin activity or hypofibrinolysis, associated with thrombosis (Moore et al. 2015; Raza et al. 2013; Schochl et al. 2012).

Recent investigations have demonstrated that plasmin, in addition to its canonical role of degrading fibrin, is also essential for musculoskeletal tissue repair (Mignemi et al. 2017; Roth et al. 2006; Schafer et al. 1994; Suelves et al. 2002; Sulniute et al. 2016). The purpose of this work was to develop and validate a pre-clinical murine model of elbow stiffness, resulting from peri-elbow soft tissue injury, with features characteristic of pathology observed in patients. Given that both the degree of elbow stiffness (Brinsden et al. 2008; King and Faber 2000; Mansat et al. 2000) and plasmin activity reduction (Amaro et al. 2017; Gibson et al. 2017) have been related to the severity of injury, we examined if a focal peri-elbow soft tissue injury, in conjunction with an investigator-imposed plasminogen deficiency, was sufficient to model trauma-induced elbow stiffness.

## Methods

### Murine model of Peri-articular injury to the elbow

All animal procedures were approved by the Vanderbilt University Institutional Animal Care and Use Committee (M1600225) and carried out in strict accordance with the recommendation in the Guide for the Care and Use of Laboratory Animals of the National Institutes of Health. All skeletal muscle injuries were performed under anesthesia, and all efforts were made to minimize suffering.

**Fig. 1:**
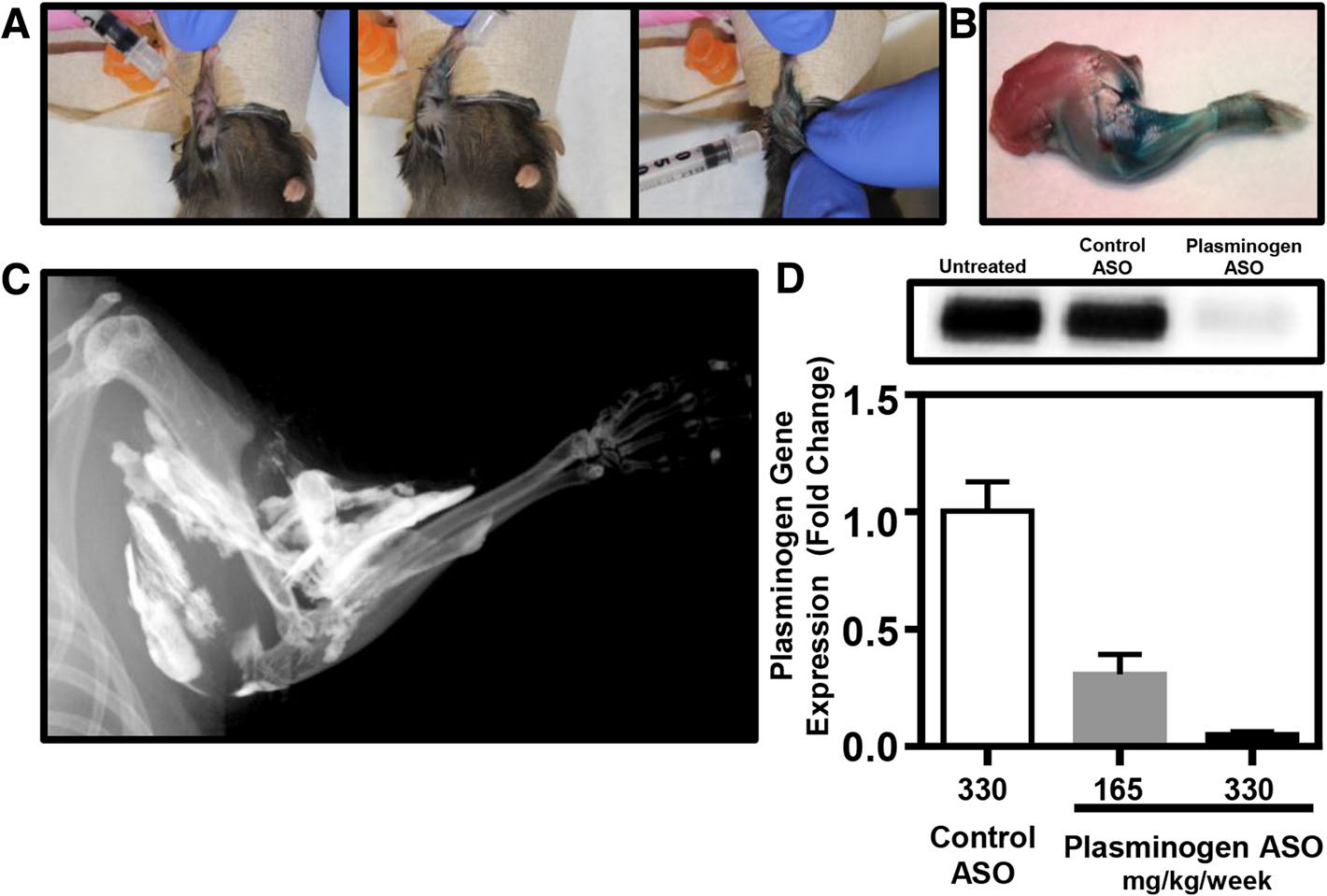
Preclinical Model of Upper Extremity Injury. **a** To reliably induce soft tissue injury around the elbow, three cardiotoxin injections were applied to the triceps, brachialis/brachioradialis, and the pronosupinators/hand extrinsics. **b** Injection of either blue dye or **c**) barium sulfate solution with subsequent radiographic analysis demonstrated well dispersed injection areas, fully surrounding the elbow joint and adjacent soft tissue. **d** Investigator induce plasminogen deficiency is attained by the time of upper extremity injury on both a protein (inset box) and RNA level following weekly administration of plasminogen ASO (330 mg/kg/wk) beginning two weeks before injury. Graphical representation of mean +/−SD

Male C57BL/6 J mice were purchased from Jackson Laboratory and housed at Vanderbilt University in a 12-h light/dark cycle with food and water provided ad libitum. At 6 weeks of age, a cardiotoxin-induce injury was administered to the soft tissues surrounding the elbow (Garry et al. 2016; Mignemi et al. 2017; Moore et al. 2016). Following anesthetization with Isoflurane, mice were placed in the Trendelenburg position, supinating the forearm, while flexing the forearm over the surface of the nose cone (Fig. 1a). From this position, 20 μL of 10 μM cardiotoxin was injected with a 28.5 G 0.5 mL insulin syringe into the posterior compartment of the arm to infiltrate the elbow extensors (triceps), the lateral compartment of the arm/forearm to infiltrate the elbow flexors (brachialis) and mobile wad (brachioradialis), and the medial aspect of the proximal forearm (pronosupinators and hand extrinsics), resulting in a total of three injection regions (20 μL each) surrounding the elbow joint (Fig. 1b and c). Both the left and right upper limbs were injured.

To induce an investigator-imposed plasminogen deficiency, a plasminogen specific antisense oligonucleotide (ASO) or a non-targeting control ASO was injected subcutaneously (330 mg/kg/week) beginning two weeks before injury and continuing through the duration of the study (Fig. 1d) (Mignemi et al. 2017). All antisense oligonucleotides used in this study were developed in collaboration with Ionis Pharmaceuticals (Carlsbad, CA).

### Quantification of elbow function following Peri-articular injury

To assess changes in elbow function following peri-articular injury, grip strength and gait analysis were performed 28 days following injury. Grip strength was assessed with an animal grip strength system force meter (San Diego Instruments, San Diego, CA). Briefly, a mouse is placed on the wire grid and allowed to grab on with its forepaws. Once secure, the mouse’s tail is gently pulled backwards and the maximum force of the grip is recorded in Newtons. This test was performed three times per mouse with 5–10 min of rest between measurements. The data from each trial is then averaged before the final analysis between experimental groups. Gait analysis was assessed with a Treadscan System (Clever Sys Inc. Reston, VA) to evaluate changes in gait disturbances. Briefly, mice were placed in a clear acrylic box above a treadmill monitored by a high-speed camera. The treadmill was then equilibrated to a speed of 13.7 cm/s and a 20 s video of the mouse’s walking pattern was obtained. Tredscan Software was then utilized to assess active range of motion in the longitudinal direction per limb. Results are presented as a mean step distance (mm) per animal.

### Histological analysis

Following sacrifice at 28 days post peri-articular injury, the upper extremity was disarticulated at the glenohumeral joint, fixed in 10% neutral buffered formalin, decalcified in 0.5 M EDTA for one week, processed, and embedded in paraffin prior to frontal or transverse sectioning. Six micron frontal section of the upper extremity were produced and stained with hematoxylin and eosin (H/E) and Martius Scarlet Blue (MSB). Additionally, at the time of sacrifice, the triceps were dissected, fixed in 10% Neutral buffered formalin, processed, and embedded in paraffin prior to transverse sectioning. Six micron sections were then stained with H/E, MSB, or Von Kossa to visualize deposits of calcification. Additionally, immunofluorescence and immunohistochemistry were performed to detect the presence of fibrin or F4/80+ cells within damaged tissues, respectively. Whole slide imaging of frontal sections was performed in the Digital Histology Shared Resource at Vanderbilt University.

Hematoxylin and Eosin (H/E) staining was performed according to standard protocols to assess tissue morphology as previously described(Mignemi et al. 2017; Moore et al. 2016). Martius Scarlet Blue (MSB) staining was performed according to standard protocols to assess for the presence of fibrin and collagen deposits within damaged tissues. Von Kossa staining to assess calcific deposits was performed according to standard protocols as previously described. (Mignemi et al. 2017; Moore et al. 2016) Immunohistochemical staining of F/480+ cells were performed according to standard protocols by the Vanderbilt University Medical Center Tissue Pathology Shared Resource. Immunofluorescent staining for the presence of fibrin was performed as previously described (Mignemi et al. 2016).

### Quantification of heterotopic ossification

Longitudinal radiographic analysis was performed weekly following injury to visualize and quantify heterotopic ossification within the injured tissues of the upper extremity. Briefly, following adequate anesthesia, digital radiographs (Faxitron LX60, Tucson, AZ) were collected weekly at an exposure of 35 kV for 4 s. Digital radiographs were standardized and the extent of muscle calcification was quantified using a modified version of the Soft Tissue Calcification Scoring System (STiCSS) for the upper extremity (Additional file 1: Figure S1) (Moore et al. 2016). All radiographic images were scored in a blinded manner by three independent observers found to be in substantial to almost perfect agreement (Kappa = 0.706–0.901, Additional file 2: Table S1) per the Landis and Koch criteria (Cohen 1968). As such, results reported represent the total score for a single limb (either left or right) as scored by a single observer.

In addition to radiographic analysis, microcomputed tomography (μCT) was utilized to visualize the formation of soft tissue calcification around the elbow joint. μCT images were acquired (μCT 40, Scanco Medical AG, Bassersdorf Switzerland) of injured forelimbs at 55kVp, a45uA, 200 ms integration, 500 projections per 180-degree rotation with a 20 μm isotropic voxel size. After scanning, the volume of interest containing the entire forelimb was selected the calcified tissues were segmented form soft tissues using a threshold of 220 per thousand (or 450.7mgHA/cm^3^), a Gaussian noise filter of 0.2, and support of 1.

### Statistical analysis

Statistical analyses of functional changes between groups were evaluated using an ordinary two-way ANOVA with a multiple comparison test. *P* values reported are adjusted for multiplicity. STiCSS upper extremity scores were statistically evaluated between groups using a non-parametric Mann-Whitney test. For all analysis, alpha = 0.05.

## Results

### Peri-elbow soft tissue injury, in conjunction with an investigator-imposed plasminogen deficiency, results in a significant loss of elbow function

**Fig. 2:**
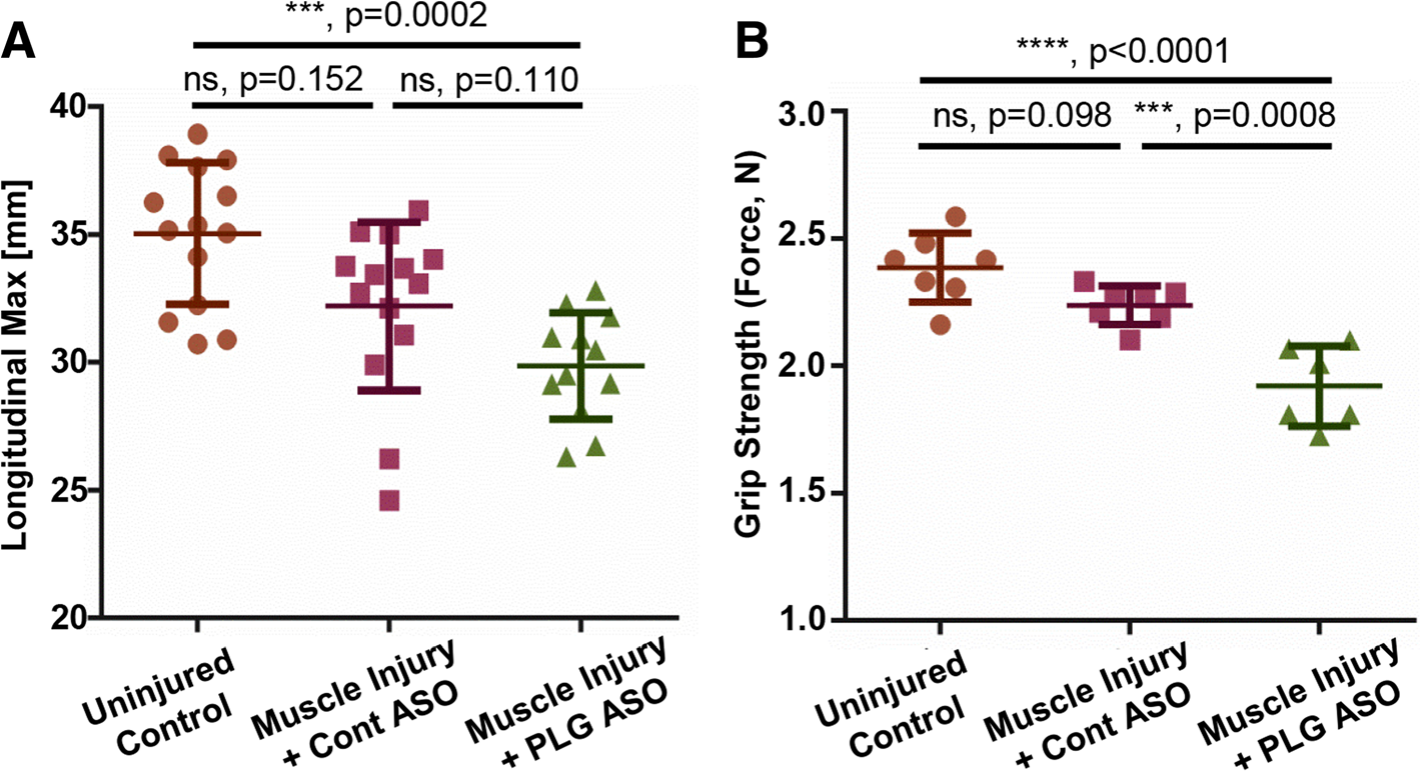
Upper extremity functional assessment following per-elbow soft tissue injury. To assess changes in upper extremity function in our model following injury with or without an investigator induce plasminogen (PLG) deficiency, **a** Treadscan analysis was utilized to measure active motion in longitudinal direction. Points (n) represent individual limbs, left and right per mouse. *N* = 7; uninjured controls, 7; Injury + Control ASO, 7; Injury + PLG ASO. *n* = 14 points per group. Mean ± SD: Uninjured control- 35.05 ± 2.776; Muscle Injury + Control ASO- 32.20 ± 3.294; Muscle Injury + PLG ASO- 29.87 ± 2.075. **b** Grip strength analysis plotted as an average per mouse. *N* = 7; uninjured controls, 7; Injury + Control ASO, 7; Injury + PLG ASO. Total of 7 data points per group. Mean ± SD: Uninjured control- 2.387 ± 0.136; Muscle Injury + Control ASO- 2.238 ± 0.076; Muscle Injury + PLG ASO- 1.921 ± 0.157. Data represented in all plots as mean +/− SD. Statistical difference was assessed by an ordinary two-way ANOVA with a Tukey’s multiple comparison test. *P* values reported are adjusted for multiplicity

Given that functional deficits following a peri-articular injury are a life altering event, we first assessed in our murine model if following a peri-elbow injury, in conjunction with an investigator-induced plasminogen deficiency, we could detect a change in upper extremity function. At 28 days post injury (DPI), we observed marked functional changes for both active longitudinal motion (Fig. 2a) and grip strength (Fig. 2b) in plasminogen deficient animals as compared to either uninjured mice or injured + control ASO treated animals. Interestingly, we also observed marked functional changes between uninjured control and injured + control ASO treated animals, indicating that peri-elbow injury alone, independent of plasminogen deficiency, was sufficient to impact elbow function. Taken together, these results demonstrated that an investigator-induced plasminogen deficiency, in conjunction with a focal peri-elbow soft tissue injury, is sufficient to impact elbow function.

### Peri-elbow soft tissue injury, in conjunction with an investigator-imposed plasminogen deficiency, results in delayed muscle repair, fibrosis, and chronic inflammation

**Fig. 3:**
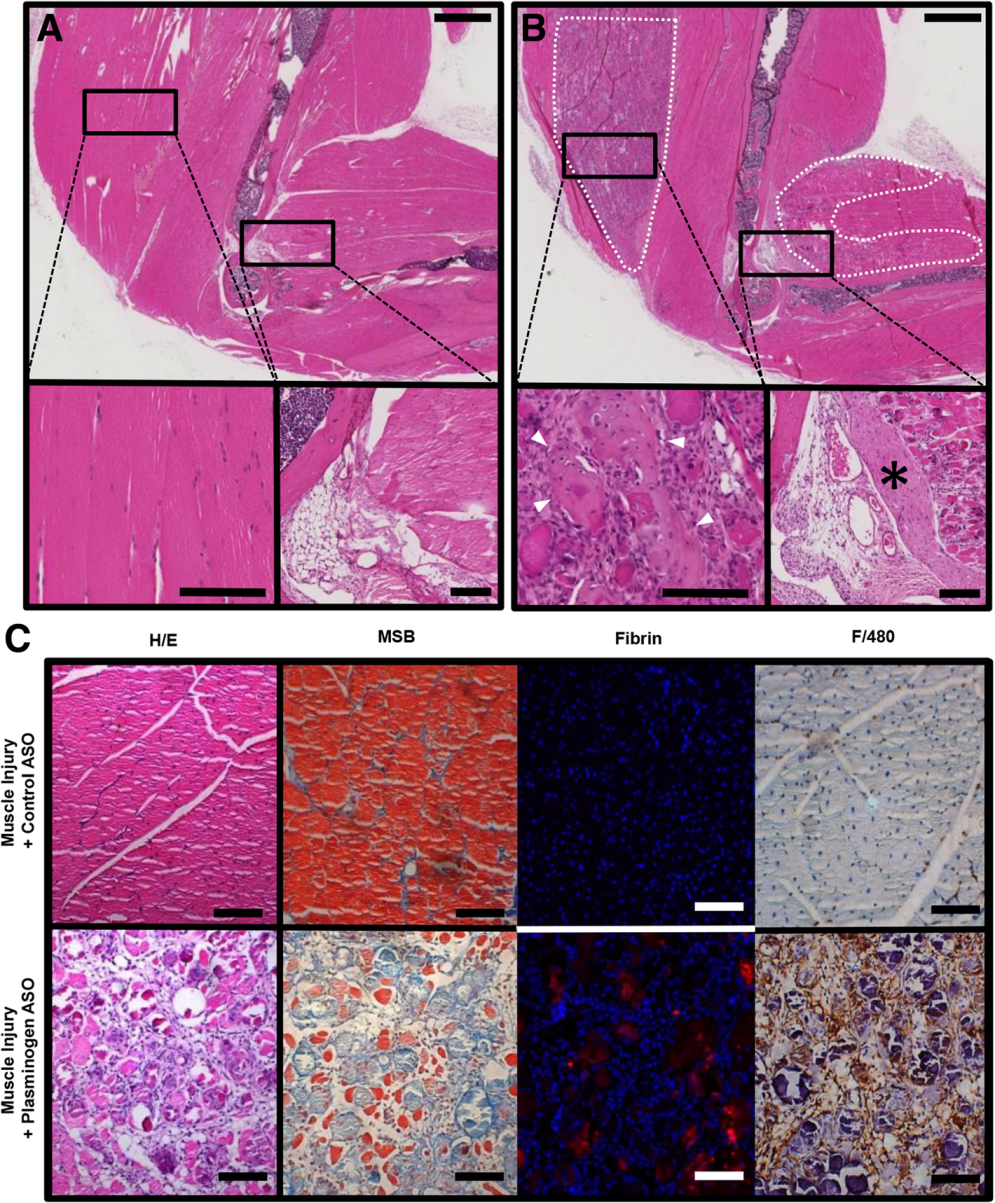
Histological analysis of frontal and transverse sections of injured peri-elbow soft tissues 28 DPI. H/E staining of frontal sections from **a**) Control ASO or **b**) plasminogen ASO treated animals. Gross morphology of whole limb at 1×, scale bar represents 1 mm. Zoomed in sections of the injured triceps muscle or capsule immediately adjacent to the capitulum and radial head (20× and 10× respectively, scale bar represents 100 μm). Black asterisk indicates thickening of the capsule observed in plasminogen ASO treated animals. White arrows indicate focal chondrocytic lesions indicative of heterotopic ossification formation. **c** Transverse sections from control ASO or plasminogen ASO treated animals, stained with H/E, MSB, immunofluorescent staining for fibrin(ogen), or immunohistochemical staining for F4/80+ cells (macrophage/monocytes). All transverse images 20× magnification, scale bar represents 100 μm

To assess soft tissue healing following peri-elbow injury, in conjunction with an investigator-imposed plasminogen deficiency, the upper extremity was isolated at 28 DPI and sectioned in either the frontal and transverse plain for histological analysis. Hematoxylin and eosin (H/E) staining of frontal sections from control ASO (Fig. 3) treated animals demonstrated normal skeletal muscle regeneration and soft tissue architecture. Alternatively, mice with an investigator-imposed plasminogen deficiency (PLG ASO treated) (Fig. 3b), possessed substantial areas of disorganized necrotic sarcomeres characterized by hypereosinophilic staining, hyalinized cytoplasm, and absent nuclei (see white dashed outline). Additionally, plasminogen deficient animals also possessed marked thinking of the capsule immediately adjacent to the capitulum and radial head (Fig. 3b- black asterisk) as well as chondrocyte laden lesions, characteristic of early heterotopic ossification (Fig. 3b- white arrow).

Further histological analysis of transverse sections (Fig. 3c) demonstrated substantial deposits of connective tissues or fibrosis, as demonstrated by MSB staining, while immunofluorescent staining for fibrin(ogen) demonstrated marked co-localization to areas of necrotic sarcomeres in plasminogen deficient animals. Furthermore, immunohistochemical staining detected substantial F4/80+ cellular infiltrate surrounding necrotic sarcomeres in plasminogen deficient animals, but limited F4/80+ cells within regenerated sarcomeres of control ASO treated animals. Taken together, these results indicated that following peri-elbow injury, an investigator-induced plasminogen deficiency, is sufficient to induce characteristically similar impaired soft tissue healing seen clinically in patients following traumatic elbow injuries.

### Peri-elbow soft tissue injury, in conjunction with an investigator-induced plasminogen deficiency, results in heterotopic ossification surrounding the elbow

**Fig. 4:**
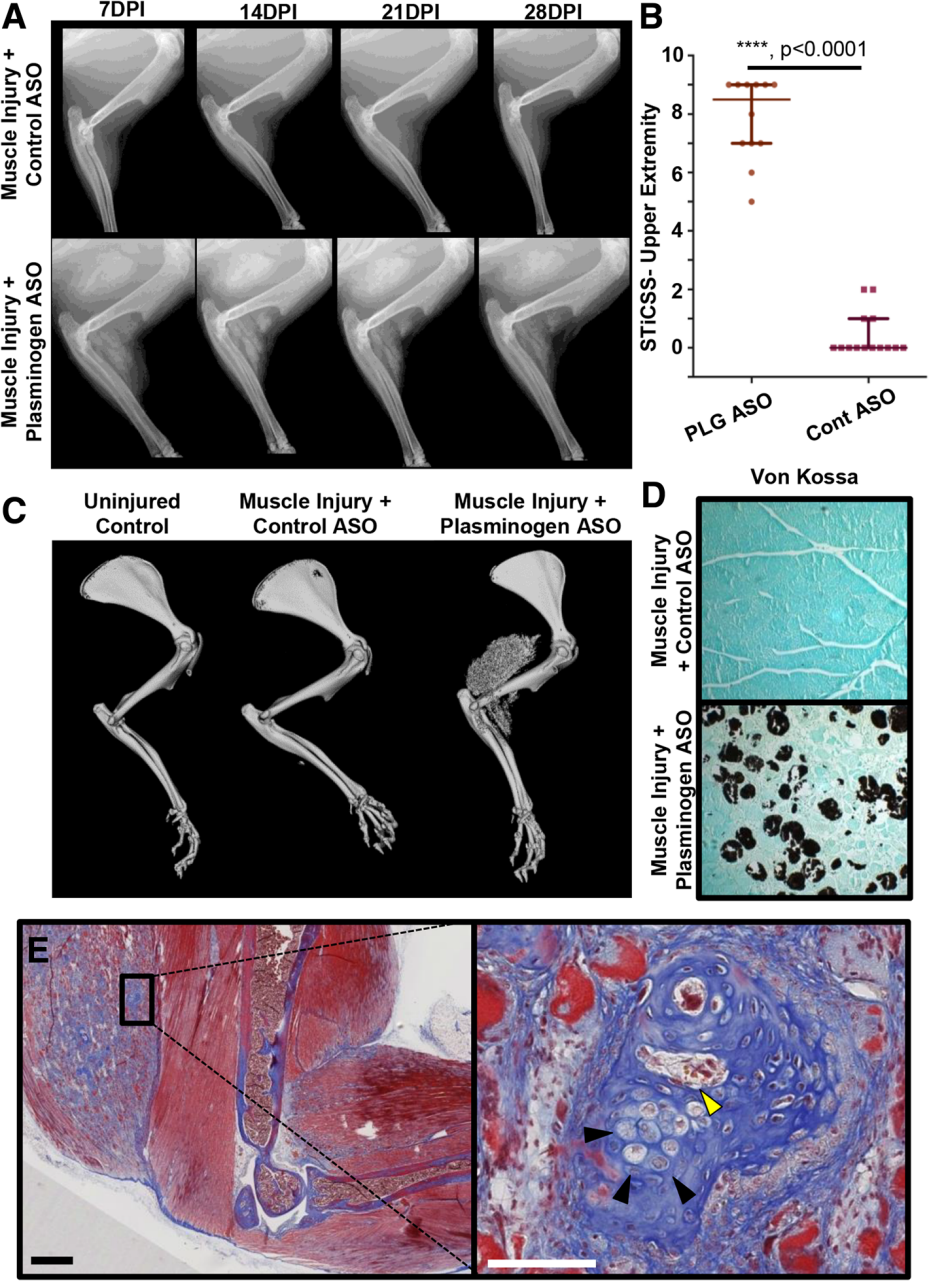
Characterization of skeletal muscle calcification following peri-elbow injury. **a** Longitudinal radiographic analysis of control ASO or plasminogen (PLG) ASO treated mice at 7, 14, 21, and 28 DPI. **b** Quantification of the soft tissue calcification within the triceps and muscle of the forearm as scored by the modified STiCSS for upper extremity. Error bar represent median and interquartile range. Statistical analyses between groups were analyzed by a non-parametric Mann-Whitney test. ****, *p* < 0.0001. **c** μCT analysis and 3D reconstruction of uninjured and injured upper extremities treated with control ASO or plasminogen ASO. **d** Von kossa staining of transvers sections of injured triceps 28 days post injury. **e** Gross morphology of PLG ASO treated animals at 1× (scale bar represents 1 mm) stained with MSB. **b** Zoomed in section of the injury triceps (20×, scale bar represents 100 μm). Black arrow heads indicate hypertrophic chondrocytes. Yellow arrow heads indicate erythrocytes

Next, we further assessed the formation of heterotopic ossification following peri-elbow injury, in conjunction with an investigator-imposed plasminogen deficiency, at 28 DPI. Longitudinal radiographic, μCT analysis, and 3D reconstruction demonstrated significantly greater amounts of heterotopic ossification surrounding the elbow of plasminogen ASO treated animals compared to control ASO treated animals (Fig. 4a-c). Furthermore, detailed histological analysis of the injured skeletal muscle stained positive for the presence of calcium as indicated by black coloration (Fig. 4d) and possessed regions with distinctive patterning of heterotopic ossification inducing the presence of hypertrophic chondrocytes (Fig. 4e- black arrows) and early hematopoiesis, as indicated by yellow stained erythrocytes (Fig. 4e- yellow arrow). Taken together, these results indicate that an investigator-imposed plasminogen deficiency, in combination with a peri-elbow soft tissue injury, results in the development of heterotopic bone that progresses through endochondral ossification.

## Conclusion & discussion

Through this study, we successfully developed and validated a novel murine model which, following a peri-elbow tissue injury in conjunction with an investigator-imposed reduction of plasminogen, recapitulates features characteristic of pathology observed in patients clinically. This model fills a necessary gap for basic science and translational studies to investigate trauma-induce elbow stiffness. Specifically, this model will be valuable for assessing novel pharmacologic treatments, given that current therapeutics including anti-inflammatory agents, pyrophosphate analogues, ionizing radiation therapy, BMP antagonists, and retinoic acid receptor agonists (Evans et al. 2009), have been demonstrated to possess negative side effects, in particular to fracture healing. Given that many times elbow stiffness occurs in combination with other traumatic injuries, current therapeutic measures effectively sacrifice the healing of one tissue (bone) for proper healing of the other (soft tissues/skeletal muscle). With this new model, we now can assess novel therapeutics aimed at preventing trauma-induced elbow stiffness, while simultaneously working to preserve bone formation and regeneration.

Previous investigations have demonstrated the plasmin is an essential reparative protease, which in addition to its canonical function of degrading fibrin, also acts on growth factors, extracellular matrix proteins, and other protease zymogens (Kanno et al. 2006; Khalil et al. 1996; Ploplis et al. 1998; Roth et al. 2006; Schoenecker et al. 2010; Yee et al. 1993). Following severe injury, it has been well demonstrated independently that plasmin activity is reduced and elbow stiffness can develop. Furthermore, both the degree of elbow stiffness (Brinsden et al. 2008; King and Faber 2000; Mansat et al. 2000) and reduction of plasmin activity (Amaro et al. 2017; Gibson et al. 2017) have been individually related to the severity of injury. However, to date it has yet to be determined clinically if these two events are related. Through the development of this model, we observed that a focal peri-elbow soft tissue injury, in conjunction with an investigator-imposed plasminogen deficiency, was sufficient to model trauma-induced elbow stiffness. Importantly, unlike genetically plasminogen deficient mice that possess impaired growth, develop rectal prolapses, and die prematurely (Ploplis et al. 1995), use of a plasminogen-targeted ASO permits temporally-controlled reduction of plasminogen, thereby allowing mice to develop normally and avoid premature death. While no adverse side effects have been observed by our laboratory following the administration of plasminogen ASO (Mignemi et al. 2017; Moore et al. 2016; Yuasa et al. 2015), given that the ASOs developed primarily target the liver where plasminogen is produced, high dose administration of ASOs can result in liver toxicity and should be considered when designing similar studies. Taken together, these results support the hypothesis that plasmin may play a role in peri-elbow tissue repair and elbow function. Strengthened by the availability of critical experimental tools (i.e. plasminogen-targeted ASO) and a novel murine model, this manuscript provides the foundation for further studies assessing i) the molecular mechanisms of plasminogen as it relates trauma-induced elbow stiffness and ii) the clinical correlations between changes in plasmin activity following injury and resulting elbow stiffness.

Akin to our findings within, recently a rat model of post-traumatic elbow contracture was developed and demonstrated to possess persistent joint motion loss and increased capsule thickening. This model, which utilizes focal peri-elbow soft tissue damage and subsequent immobilization to induce elbow stiffness is, to our knowledge, the only other small animal model of elbow stiffness currently reported (Dunham et al. 2017a; Dunham et al. 2017b; Lake et al. 2016). Here, in our murine model, we observed loss of joint function and capsule thickening (as reported in the rat model above), as well as tissue scaring, fibrosis, and of the formation of heterotopic ossification; all pathologies suggested clinically to impact elbow stiffness. Yet, this model is not without limitations. Specifically, the cardiotoxin injury method cannot localize the injury to a single type of soft tissue, therefore we cannot partition plasmin’s role in skin, tendon, skeletal muscle, and capsule healing individually as they pertain to elbow stiffness. Additionally, this study did not utilize post-operative immobilization of the affected limb, yet this current model could be utilized to examine such a hypothesis. Finally, while the elbow is highly susceptible to impaired function and stiffness following traumatic injury, we hypothesize that this model has the potential to be translated to other joints, such as the knee, hip, or shoulder, given that each of these joints have been found clinically to be susceptible to pathologic calcification, fibrosis, and impaired soft tissue healing following injury.

## Additional files

Additional file 1: **Figure S1.** Quantification of muscle calcification by radiographic analysis. To quantify muscle calcification, digital radiographs were first standardized using ImageJ (Step 1: Image Processing) by adjusting the minimum and maximum brightness of the image so that no soft tissue is visible on the extensor surface or the distal half of the radius. Next, processed digital radiographs were scored by an ordinal scale of 0–3 represent varying degrees of calcification with a score of “0” indicating no visible calcification, “1” indicating trace amounts of calcification with < 10% of the region of interest being calcified, “2” indicating moderate calcification with 10–50% of the region of interest being calcified, and “3” indicating severe soft tissue calcification with > 50% of the region of interest being calcified. Given the multiple soft tissue injury method, we assessed the development of calcification in three distinct anatomical locations (the biceps, triceps, and proximal forearm), assigned a score to each area, and reported the final score per animals as a sum of the individual scores. Therefore, the highest possible score is 9. (TIF 1159 kb)

Additional file 2: **Table S1.** Interobserver agreement for quantification of soft tissue calcification surrounding the elbow. (DOCX 13 kb)

## Abbreviations

ANOVA: Analysis of variance
ASO: Antisense oligonucleotide
BMP: Bone morphogenic protein
DPI: Days post injury
H/E: Hematoxylin and Eosin
MSB: Martius Scarlet Blue
PLG: Plasminogen
STiCSS: Soft Tissue Calcification Scoring System
μCT: Microcomputed tomography

## References

[CR1] Agarwal S, Loder S, Levi B (2017). Heterotopic ossification following upper extremity injury. Hand Clin.

[CR2] Amaro E (2017). Abstract P20: severe injury leads to plasmin consumption below a critical threshold required to heal soft tissue injury. Plastic and Reconstructive Surgery – Global Open.

[CR3] Brinsden MD, Carr AJ, Rees JL (2008). Post-traumatic flexion contractures of the elbow: operative treatment via the limited lateral approach. J Orthop Surg Res.

[CR4] Cohen J (1968). Weighted kappa: nominal scale agreement with provision for scaled disagreement or partial credit. Psychol Bull.

[CR5] Djurickovic S, Meek RN, Snelling CF, Broekhuyse HM, Blachut PA, O'Brien PJ, Boyle JC (1996). Range of motion and complications after postburn heterotopic bone excision about the elbow. J Trauma.

[CR6] Dunham Chelsey L., Castile Ryan M., Chamberlain Aaron M., Galatz Leesa M., Lake Spencer P. (2017). Pronation–Supination Motion Is Altered in a Rat Model of Post-Traumatic Elbow Contracture. Journal of Biomechanical Engineering.

[CR7] Dunham CL, Castile RM, Havlioglu N, Chamberlain AM, Galatz LM, Lake SP (2017). Persistent motion loss after free joint mobilization in a rat model of post-traumatic elbow contracture. J Shoulder Elb Surg.

[CR8] Evans PJ, Nandi S, Maschke S, Hoyen HA, Lawton JN (2009). Prevention and treatment of elbow stiffness. J Hand Surg Am.

[CR9] Garland DE, O'Hollaren RM (1982). Fractures and dislocations about the elbow in the head-injured adult. Clin Orthop Relat Res.

[CR10] Garry GA, Antony ML, Garry DJ (2016). Cardiotoxin induced injury and skeletal muscle regeneration. Methods Mol Biol.

[CR11] Gibson B, Moore-Lotridge S, Mignemi N, Hawley G, Oelsner W, Schoenecker J (2017) The Consumption of Plasminogen Following Severe Burn and Its Implications in Muscle Calcification The FASEB Journal 31:390.394–390.394 doi:10.1096/fasebj.31.1_supplement.390.4

[CR12] Josefsson PO, Johnell O, Gentz CF (1984). Long-term sequelae of simple dislocation of the elbow. J Bone Joint Surg Am.

[CR13] Kanno Y (2006). Lack of alpha2-antiplasmin improves cutaneous wound healing via over-released vascular endothelial growth factor-induced angiogenesis in wound lesions. J Thromb Haemost.

[CR14] Khalil N, Corne S, Whitman C, Yacyshyn H (1996). Plasmin regulates the activation of cell-associated latent TGF-beta 1 secreted by rat alveolar macrophages after in vivo bleomycin injury. Am J Respir Cell Mol Biol.

[CR15] King GJ, Faber KJ (2000). Posttraumatic elbow stiffness. Orthopedic Clinics.

[CR16] Lake SP, Castile RM, Borinsky S, Dunham CL, Havlioglu N, Galatz LM (2016). Development and use of an animal model to study post-traumatic stiffness and contracture of the elbow. J Orthop Res.

[CR17] Mansat P, Morrey B, Hotchkiss R (2000) Extrinsic contracture:" the column procedure," lateral and medial capsular releases The elbow and its disorders

[CR18] Mignemi Nicholas A, Yuasa Masato, Baker Courtney E, Moore Stephanie N, Ihejirika Rivka C, Oelsner William K, Wallace Christopher S, Yoshii Toshitaka, Okawa Atsushi, Revenko Alexey S, MacLeod A Robert, Bhattacharjee Gourab, Barnett Joey V, Schwartz Herbert S, Degen Jay L, Flick Matthew J, Cates Justin M, Schoenecker Jonathan G (2016). Plasmin Prevents Dystrophic Calcification After Muscle Injury. Journal of Bone and Mineral Research.

[CR19] Mignemi NA (2017). Plasmin prevents dystrophic calcification after muscle injury. J Bone Miner Res.

[CR20] Moore EE (2015). Postinjury fibrinolysis shutdown: rationale for selective tranexamic acid. J Trauma Acute Care Surg.

[CR21] Moore SN (2016). Validation of a radiography-based quantification designed to longitudinally monitor soft tissue calcification in skeletal muscle. PLoS One.

[CR22] Morrey BF, Askew LJ, Chao EY (1981). A biomechanical study of normal functional elbow motion. J Bone Joint Surg Am.

[CR23] Ploplis VA, Carmeliet P, Vazirzadeh S, Van Vlaenderen I, Moons L, Plow EF, Collen D (1995). Effects of disruption of the plasminogen gene on thrombosis, growth, and health in mice. Circulation.

[CR24] Ploplis VA, French EL, Carmeliet P, Collen D, Plow EF (1998). Plasminogen deficiency differentially affects recruitment of inflammatory cell populations in mice. Blood.

[CR25] Raza I (2013). The incidence and magnitude of fibrinolytic activation in trauma patients. J Thromb Haemost.

[CR26] Roth D (2006). Plasmin modulates vascular endothelial growth factor-A-mediated angiogenesis during wound repair. Am J Pathol.

[CR27] Schafer BM, Maier K, Eickhoff U, Todd RF, Kramer MD (1994). Plasminogen activation in healing human wounds. Am J Pathol.

[CR28] Schochl H, Voelckel W, Maegele M, Solomon C (2012). Trauma-associated hyperfibrinolysis. Hamostaseologie.

[CR29] Schoenecker J (2010). 2010 young investigator award winner: therapeutic aprotinin stimulates osteoblast proliferation but inhibits differentiation and bone matrix mineralization. spine (Phila pa 1976).

[CR30] Seth MK, Khurana JK (1985). Bony ankylosis of the elbow after burns. J Bone Joint Surg Br.

[CR31] Suelves M (2002). Plasmin activity is required for myogenesis in vitro and skeletal muscle regeneration in vivo. Blood.

[CR32] Sulniute R (2016). Plasminogen is a critical regulator of cutaneous wound healing. Thromb Haemost.

[CR33] Weiss AP, Sachar K (1994). Soft tissue contractures about the elbow. Hand clinics.

[CR34] Yee JA, Yan L, Dominguez JC, Allan EH, Martin TJ (1993). Plasminogen-dependent activation of latent transforming growth factor beta (TGF beta) by growing cultures of osteoblast-like cells. J Cell Physiol.

[CR35] Yuasa M (2015). Fibrinolysis is essential for fracture repair and prevention of heterotopic ossification. J Clin Invest.

