# Supplementary figures and images for: Novel preclinical murine model of trauma-induced elbow stiffness
Source: J Exp Orthop. 2018 Sep 18;5:36. doi: 10.1186/s40634-018-0155-3 (PMC6143496; doi:10.1186/s40634-018-0155-3)

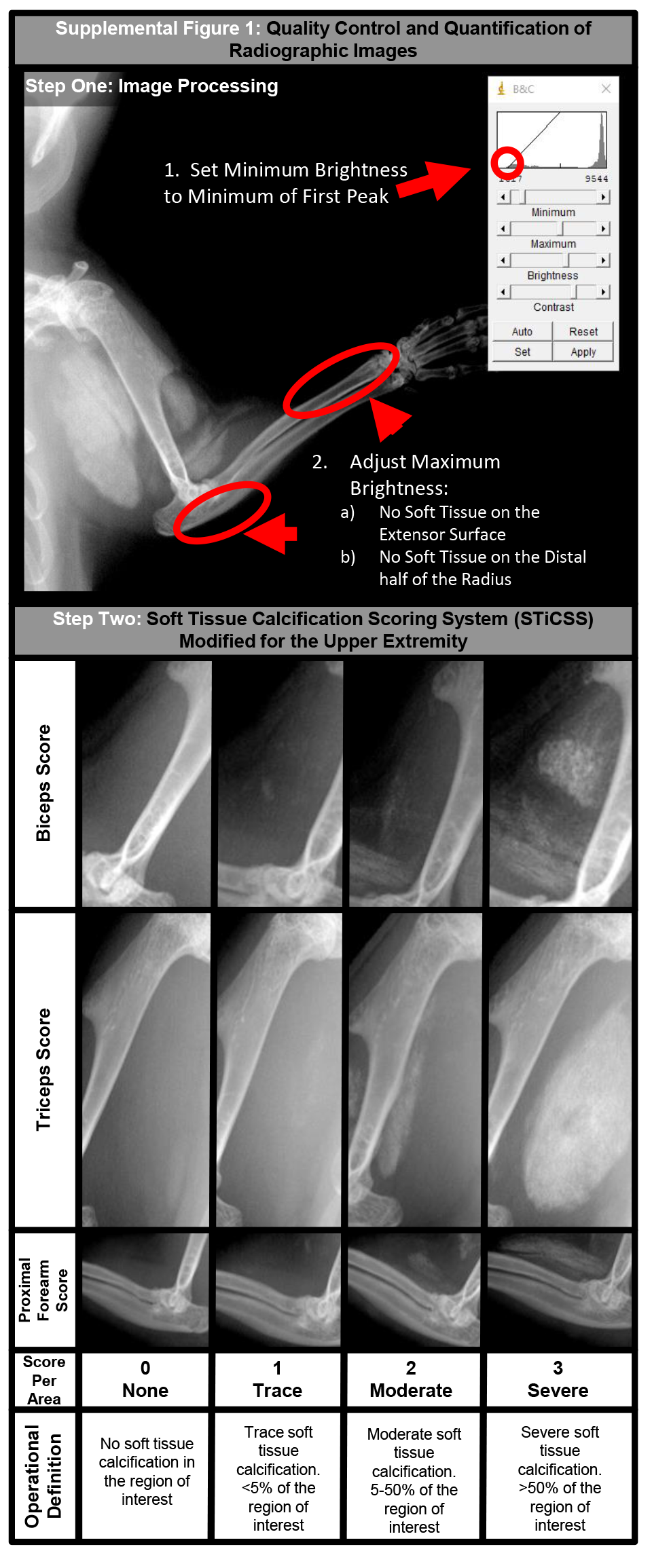

Supplement: Supplementary file 1 — Figure S1. Quantification of muscle calcification by radiographic analysis. To quantify muscle calcification, digital radiographs were first standardized using ImageJ (Step 1: Image Processing) by adjusting the minimum and maximum brightness of the image so that no soft tissue is visible on the extensor surface or the distal half of the radius. Next, processed digital radiographs were scored by an ordinal scale of 0–3 represent varying degrees of calcification with a score of “0” indicating no visible calcification, “1” indicating trace amounts of calcification with < 10% of the region of interest being calcified, “2” indicating moderate calcification with 10–50% of the region of interest being calcified, and “3” indicating severe soft tissue calcification with > 50% of the region of interest being calcified. Given the multiple soft tissue injury method, we assessed the development of calcification in three distinct anatomical locations (the biceps, triceps, and proximal forearm), assigned a score to each area, and reported the final score per animals as a sum of the individual scores. Therefore, the highest possible score is 9. (TIF 1159 kb) [file 40634_2018_155_MOESM1_ESM.tif]
