# Supplementary material for: Novel preclinical murine model of trauma-induced elbow stiffness
Source: J Exp Orthop. 2018 Sep 18;5:36. doi: 10.1186/s40634-018-0155-3 (PMC6143496; doi:10.1186/s40634-018-0155-3)
Supplement: Supplementary file 2 — Table S1. Interobserver agreement for quantification of soft tissue calcification surrounding the elbow. (DOCX 13 kb) [file 40634_2018_155_MOESM2_ESM.docx]

**Table S1: Interobserver agreement for quantification of soft tissue calcification surrounding the elbow.**

|  | **A** | **B** | **C** |
| --- | --- | --- | --- |
| **A** |  | 0.901 | 0.706 |
| **B** |  |  | 0.756 |
| **C** |  |  |  |
